# Editorial: Molecular mechanisms and targeted therapies for colorectal cancer vol II

**DOI:** 10.3389/fonc.2025.1566681

**Published:** 2025-02-18

**Authors:** Alessandro Passardi, David Gibbons, Antonio Mario Scanu, Shilpa S Dhar

**Affiliations:** ^1^ Department of Medical Oncology, IRCCS Istituto Romagnolo per lo Studio dei Tumori (IRST) “Dino Amadori”, Meldola, Italy; ^2^ Department of Histopathology, St. Vincent’s University Hospital, Dublin, Ireland; ^3^ The School of Medicine and Health Sciences, University College, Dublin, Ireland; ^4^ Department of Medicine, Surgery and Pharmacy, Unit of General Surgery 2, University of Sassari, Sassari, Italy; ^5^ Department of Gastrointestinal Medical Oncology, The University of Texas MD Anderson Cancer Center, Houston, TX, United States

**Keywords:** colorectal cancer, colorectal liver metastasis, immunocheck point inhibitors, molecular marker, early diagnosis

Colorectal cancer (CRC) remains a major global health challenge, ranking as the third most common cancer and causing over 500,000 deaths annually. Despite advancements in treatments such as surgery, chemotherapy, radiotherapy, and targeted therapies—including anti-VEGF and anti-EGFR agents—the 5-year survival rate for metastatic CRC remains low, at approximately 35–40% (1, 2). This underscores the urgent need for deeper understanding and innovative approaches. Metastasis, particularly to the liver—which occurs in nearly half of CRC cases—is a critical factor in reduced survival rates, highlighting the necessity of investigating the molecular mechanisms that drive tumor proliferation, migration, and invasion.

Understanding the factors contributing to CRC is crucial for its prevention and control. However, the drivers behind its rising incidence and mortality remain unclear. Established nonmodifiable risk factors include age, male sex, and family history, while modifiable factors such as smoking, alcohol consumption, obesity, and red meat intake also play significant roles. Identifying additional modifiable risk factors is essential for improving prevention strategies. A study by Zhou et al. employed Mendelian randomization to examine the relationship between serum uric acid (SUA) levels and the risk of colorectal, colon, and rectal cancers. While no causal association was found for colorectal or colon cancers, higher SUA levels were linked to a slightly reduced risk of rectal cancer. Sensitivity analyses supported these findings, though one genetic variant (rs1471633) influenced rectal cancer results. Further research is needed to explore the role of elevated SUA, such as in hyperuricemia or gout, in CRC development.

In addition to identifying risk factors, improving early diagnostic methods for CRC could significantly reduce mortality and alleviate the burden on patients. Peng et al. explored the diagnostic potential of serum extracellular vesicle (EV) tRF-RNAs in CRC. Among 205 CRC patients, 2 tRF-RNAs—3’tRF-ThrCGT and 3’tRF-mtlleGAT—showed significantly different expression levels compared to 201 healthy donors, with predictive diagnostic efficiencies (AUCs) of 0.669 and 0.656, respectively. Combining these markers with traditional tumor markers CEA and CA724 improved diagnostic efficiency to 0.938. These findings suggest that 3’tRF-ThrCGT and 3’tRF-mtlleGAT, together with CEA and CA724, could serve as minimally invasive biomarkers for CRC detection.

The immune system also plays a significant role in CRC development. Omran et al. investigated immune-related gene expression in CRC and adenomatous polyps within the Norwegian population to identify potential biomarkers for early detection. They analyzed 228 biopsies from 69 patients undergoing colonoscopy, divided into CRC, adenomatous polyp, and control groups. Using nCounter analysis and RT-qPCR, they examined the expression of 579 immune genes, focusing on differences between tumor and non-tumorous tissues. The results showed elevated levels of CXCL1, CXCL2, IL1B, IL6, CXCL8 (IL8), PTGS2, and SPP1 in CRC tissues. Notably, CXCL1, CXCL2, IL6, CXCL8, and PTGS2 also showed significant changes in adenomatous polyps, suggesting their involvement in early carcinogenesis. These findings highlight a unique immunological signature in colorectal neoplasia and identify these genes as promising biomarkers for CRC.

Immune checkpoint inhibitors (ICIs) have revolutionized cancer treatment by leveraging the immune system to target and destroy cancer cells. In metastatic CRC, ICIs have shown promise, particularly in specific subgroups, by improving clinical outcomes and offering new therapeutic options. Chen et al. conducted a Bayesian network meta-analysis of seven randomized controlled trials involving 1,358 patients to assess first- and second-line immunotherapy regimens for microsatellite instability (MSI) and microsatellite-stable (MSS) subgroups. Nivolumab combined with ipilimumab emerged as the most effective first-line option for MSI patients, offering significant progression-free survival (PFS) benefits (HR = 0.21) and a favorable safety profile. For MSS patients, Nivolumab showed notable PFS improvement (HR = 0.74) in first-line therapy, while Atezolizumab demonstrated efficacy in second-line treatment (HR = 0.66). These findings emphasize the clinical benefits of ICIs and their potential to inform future treatment guidelines for metastatic CRC. Third-line treatment options for metastatic CRC are varied, and ICIs may improve outcomes in this setting as well. Wu et al. evaluated the efficacy and safety of third-line therapies for refractory MSS metastatic CRC. Data from 60 patients showed that regorafenib combined with PD-1 inhibitors and trifluridine/tipiracil with bevacizumab had better PFS than fruquintinib combined with PD-1 inhibitors. However, no significant differences in overall survival were observed among the three treatments. Liver and peritoneal metastases were associated with shorter survival. Overall, regorafenib and trifluridine/tipiracil demonstrated superior PFS compared to fruquintinib.

Approximately half of CRC patients develop liver metastases, with recurrence occurring in up to 70% of cases following liver resection. Kalil et al. investigated the use of circulating tumor DNA (ctDNA) for detecting minimal residual disease (MRD) and predicting recurrence. In 29 patients with known KRAS or PIK3CA mutations, ctDNA was analyzed at 115 time points using digital PCR. Detectable ctDNA at the time of liver resection was linked to recurrence in 81% of cases, compared to 46% in those with undetectable ctDNA (p = 0.064). Postoperative ctDNA detection occurred in 27.6% of patients, all of whom later experienced radiologic recurrence, versus 52% recurrence in patients with undetectable ctDNA (p = 0.026). Detectable ctDNA postoperatively was also associated with shorter disease-free survival (9 months vs. 13 months, HR = 2.95, p = 0.02). These findings demonstrate the potential of ctDNA analysis via liquid biopsy for early recurrence detection and support its use in MRD monitoring after liver resection.

The identification of novel biomarkers and therapeutic targets is an unmet need in CRC. Liu et al. identified ZG16 as a potential therapeutic target for metastatic CRC. Using gene expression data from GEO and TCGA databases, they identified 29 hub genes, with low ZG16 expression linked to poor overall and disease-free survival. Laboratory experiments confirmed that ZG16 overexpression inhibited CRC cell proliferation, invasion, and migration while suppressing epithelial-mesenchymal transition and Wnt/β-catenin signaling. These findings suggest that ZG16 plays a critical role in CRC metastasis and could improve prognosis through targeted therapies. Finally, Meyer et al.’s study highlights the role of microRNAs (miRNAs) in CRC progression and recurrence. Analyzing 827 cancer-related miRNAs using NanoString’s nCounter technology, they identified 156 miRNAs with contrasting dysregulation patterns in recurrent tumors. Key findings included the role of the let-7 family, dysregulated target genes, and miRNAs linked to adverse outcomes. These results emphasize the potential of miRNAs as stage-specific biomarkers and therapeutic targets in CRC.
